# Nonspecific ST-T changes associated with unsatisfactory blood pressure control among adults with hypertension in China: Evidence from the CSPPT study

**DOI:** 10.1097/MD.0000000000006423

**Published:** 2017-03-31

**Authors:** Huihui Bao, Huaxiu Cai, Yan Zhao, Xiao Huang, Fangfang Fan, Chunyan Zhang, Juxiang Li, Jing Chen, Kui Hong, Ping Li, Yanqing Wu, Qinhua Wu, Binyan Wang, Xiping Xu, Yigang Li, Yong Huo, Xiaoshu Cheng

**Affiliations:** a Department of Cardiovascular Medicine, the Second Affiliated Hospital of Nanchang University, Nanchang; b Department of Cardiovascular Medicine, XinHua Hospital Affiliated to Shanghai Jiao Tong, University School of Medicine, Shanghai; c Department of Cardiology, Peking University First Hospital, Beijing; d National Clinical Research Study Center for Kidney Disease, State Key Laboratory for Organ, Failure Research, Renal Division, Nanfang Hospital, Southern Medical University, Guangzhou, China.

**Keywords:** electrocardiogram, hypertension, nonspecific ST-T changes, unsatisfactory blood pressure control

## Abstract

Nonspecific ST-segment and T-wave (ST-T) changes represent one of the most prevalent electrocardiographic abnormalities in hypertensive patients. However, a limited number of studies have investigated the association between nonspecific ST-T changes and unsatisfactory blood pressure (BP) control in adults with hypertension.

The study population comprised 15,038 hypertensive patients, who were selected from 20,702 participants in the China Stroke Primary Prevention Trial. The subjects were examined with electrocardiogram test at the initial visit in order to monitor baseline heart activity. According to the results of the electrocardiogram (defined by Minnesota coding), the subjects were divided into 2 groups: ST-T abnormal and ST-T normal. Unsatisfactory BP control was defined as systolic BP ≥140 mm Hg or diastolic BP ≥90 mm Hg following antihypertensive treatment during the 4.5-year follow-up period. Multivariate analysis was used to analyze the association between nonspecific ST-T abnormalities and unsatisfactory BP control.

Nonspecific ST-T changes were common in hypertensive adults (approximately 8.5% in the study), and more prevalent in women (10.3%) and diabetic patients (13.9%). The unsatisfactory BP control rate was high in the total population (47.0%), notably in the ST-T abnormal group (55.5%). The nonspecific ST-T abnormal group exhibited a significantly greater rate of unsatisfactory BP control (odds ratio [OR] 1.20, 95% confidence interval [CI] [1.06, 1.36], *P* = 0.005]), independent of traditional risk factors, as demonstrated by multivariate regression analysis. Notable differences were further observed in male subjects (OR 1.51, 95% CI [1.17, 1.94], *P* = 0.002) and in patients with comorbid diabetes (OR 1.47, 95% CI [1.04, 2.07], *P* = 0.029).

Greater rates of unsatisfactory BP control in hypertensive patients with electrocardiographic nonspecific ST-T abnormalities were observed, notably in the subcategories of the male subjects and the diabetic patients.

## 1 Background

Hypertension is a common chronic disease that affects a great proportion of the population worldwide. It is considered one of the major risk factors for the development of cardiovascular diseases such as stroke, ischemic heart disease, and heart failure.^[1–4]^ Despite the recent advances in the development of antihypertensive therapeutic treatments and in the pathophysiology of hypertension, the prevalence of hypertension in China remains considerably high, whereas the rates of blood pressure (BP) control are notably low. The outline of the Report on Cardiovascular Disease in China (2014) demonstrated that the hypertension control (BP <140/90 mm Hg) rate was approximately 9.3% of all patients, and/or 27.4% of treated patients, although 42.6% of hypertensive patients in China were aware of their condition and 34.1% were receiving treatment.^[5]^ Furthermore, the BP control rate was lower in female subjects and rural patients compared with that in male subjects and urban patients, respectively. The electrocardiogram (ECG) is a routine, accessible, cost-effective, and recommended diagnostic tool for the initial evaluation and follow-up of hypertensive patients. In the standard surface ECG, nonspecific ST-segment and T-wave (ST-T) changes are a common finding. Recently, a majority of studies have indicated that nonspecific ST-T abnormalities are significantly associated with cardiovascular morbidity and mortality.^[6–9]^ However, the prognostic significance of nonspecific ST-T abnormalities for BP control has not been fully investigated.

The goal of the present study was to determine whether nonspecific baseline ST-T ECG abnormalities are associated with unsatisfactory BP control in hypertensive patients. The study aimed to provide information on the potential improvement of the management of hypertension.

## 2 Methods

### 2.1 Study population

**Figure 1:**
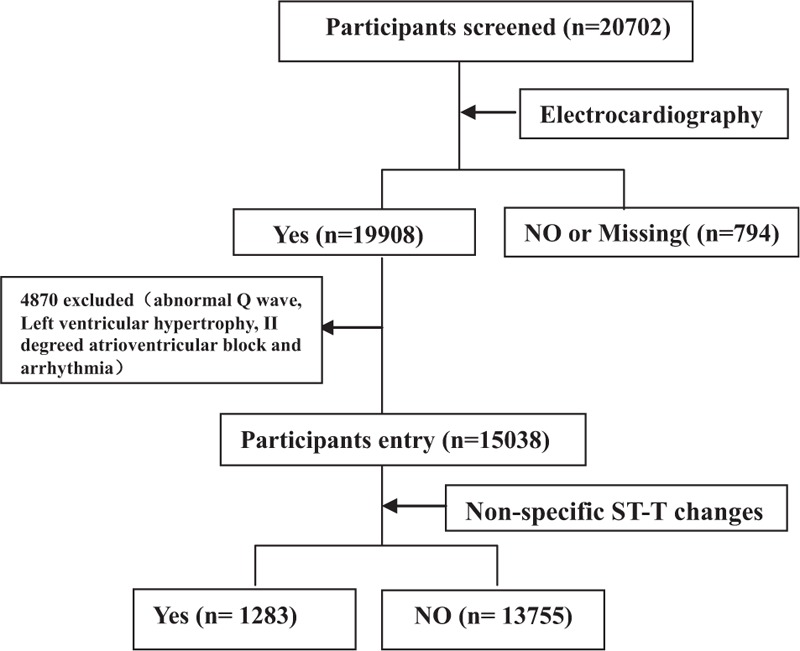
Flow of nonspecific ST-T changes in the China Stroke Primary Prevention Trial. ST-T = ST-segment and T wave.

The present study population was a subset of the China Stroke Primary Prevention Trial (CSPPT).^[2]^ The detailed description of the stage designed and the methodology have been previously described.^[2]^ Eligible participants were men and women 45 to 75 years of age who had hypertension. The major exclusion criteria included the history of stroke, myocardial infarction, heart failure, coronary revascularization, and/or congenital heart disease. Individuals with a missing ECG measurement, an illegible ECG, and/or ECG abnormalities inconsistent with nonspecific ST-T changes (abnormal Q wave, atrioventricular block, left ventricle high voltage, and arrhythmia) at baseline (Fig. 1) were further excluded. A total of 15,038 participants with hypertension were selected for the present analysis.

The CSPPT protocol was approved by the ethics committee of the Institute of Biomedicine, Anhui Medical University, Hefei, China (FWA assurance number FWA00001263). All participants provided written informed consent for their participation in the study protocol. The study protocol was conducted according to the principles of the Declaration of Helsinki that ensure that the safety and well-being of the patients is protected and that the integrity of the data is preserved.

### 2.2 Study protocol and evaluation criteria

The CSPPT was a multicommunity, randomized, double-blinded clinical trial conducted between May 19, 2008, and August 24, 2013, in 32 communities in the Jiangsu and Anhui provinces of China. The present study was a prospective cohort study. The 15,038 participants with baseline ECG measures were stratified according to the presence and/or absence of nonspecific ST-T abnormalities using the Minnesota coding criteria. Eligible participants were subsequently divided into 2 groups: ST-T abnormal and ST-T normal. All ECGs were recorded and analyzed by 2 trained medical professionals. In the case of a disagreement regarding the interpretation of an ECG, a third reading was conducted jointly until a final interpretation of the ECG was achieved.

### 2.3 Criteria for ECG Definitions

The Minnesota code (MC) criteria for nonspecific ST-T abnormalities were used, as described by the MC ECG classifications 4-3, 4-4, 5-3, and 5-4. The criteria were defined as follows: no ST-J depression ≥0.5 mm but ST-segment downward sloping and ST-segment or T-wave nadir at least 0.5 mm below the P-R baseline, in any of leads I, II, aVL, or V_2_ to V_6_ (MC 4-3); ST-J depression ≥1.0 mm and ST-segment upward sloping or U-shaped, in any of leads I, II, aVL, or V_1_ to V_6_ (MC 4-4); T-wave amplitude zero (flat), negative, or diphasic (negative–positive type only) with <1.0-mm negative phase in leads I, II, V_3_ to V_6_, aVL when R-wave amplitude is ≥5.0 mm (MC 5-3); and T-wave amplitude positive and T- to R-wave amplitude ratio of <1:20 in any of leads I, II, aVL, or V_3_ to V_6_ when R-wave amplitude in the corresponding leads was ≥10.0 mm (MC 5-4).

Hypertension was defined as seated resting systolic blood pressure (SBP) of ≥140 mm Hg or diastolic blood pressure (DBP) of ≥90 mm Hg at both the screening and recruitment visits and/or use of antihypertensive medication. The BP of the participants was measured during the follow-up period every 3 months from 2008 to 2013. During each visit, the participants were required to rest in a seated position for at least 5 minutes prior to the measurement. BP was measured 3 times at 5-minute intervals by a trained physician with an electronic sphygmomanometer (Omron; Dalian, China). The mean of the 3 readings was calculated for the measurement of the BP. The average of 20 measurements at 20 different visits was used as the final BP value. Overall unsatisfactory BP control was defined as SBP of ≥140 mm Hg or DBP of ≥90 mm Hg following antihypertensive treatment.

Diabetes mellitus was defined as self-reported clinically diagnosed diabetes or use of hypoglycemic agents or a fasting blood glucose concentration of ≥7.0 mmol/L (≥7.0 mmol/L). Body mass index (BMI) was calculated as weight (in kilograms) divided by height (in square meters).

Serum folate and vitamin B_12_ at both the baseline and the exit visits were measured by a commercial laboratory kit using a chemiluminescent immunoassay (New Industrial; Shenzhen, China). Serum homocysteine, fasting lipids, and glucose levels at the baseline and the exit visit were measured using automatic clinical analyzers (Beckman Coulter; California, America) at the core laboratory of the National Clinical Research Center for Kidney Disease, in Nanfang Hospital, Guangzhou, China.

### 2.4 Statistical analysis

Data were analyzed using the Empower Stats software. The results are presented as mean ± standard deviation for continuous variables and frequence(percentage) for categorical variables. Baseline characteristics were compared between participants in the presence and/or absence of nonspecific ST-T abnormalities using χ^2^ tests for categorical variables and *t* tests and/or Wilcoxon rank-sum tests for continuous variables, as appropriate. The prospective association between nonspecific ST-T changes and unsatisfactory BP control was examined using Cox proportional hazard regression models following adjustment for the covariates. Multiple linear regression was used to assess the association between ST-T abnormality and change of blood pressure under treatment. Furthermore, multivariate logistic regression analysis was used to evaluate the impact of electrocardiographic nonspecific ST-T abnormalities on unsatisfactory BP control in the subgroup analyses. A 2-tailed *P*-value <0.05 was considered statistically significant.

## 3 Results

The study population included 15,038 participants (62.1% women) from the CSPPT, which is representative of a low- to medium-risk Chinese hypertensive population. The BP control rate for the total population was 53.0%. The prevalence of nonspecific ST-T abnormalities in the population investigated was 8.5%, whereas prevalence was greater in women (10.3%) and diabetics (13.9%).

The baseline characteristics are shown in Table 1, according to the presence and/or absence of nonspecific ST-T abnormalities for the entire study population. The baseline characteristics were further stratified according to gender (Table 1). Following antihypertensive treatment, the ST-T abnormal group exhibited significantly greater SBP, greater change from baseline to final measurement in SBP and DBP, and a greater relative percentage decrease in both SBP and DBP from baseline to the last measurement. In addition, the unsatisfactory BP control rate was significantly greater (54.5%) in the total population, compared with that in individuals with an absence of nonspecific ST-T abnormalities (46.3%).

**Table 1:**
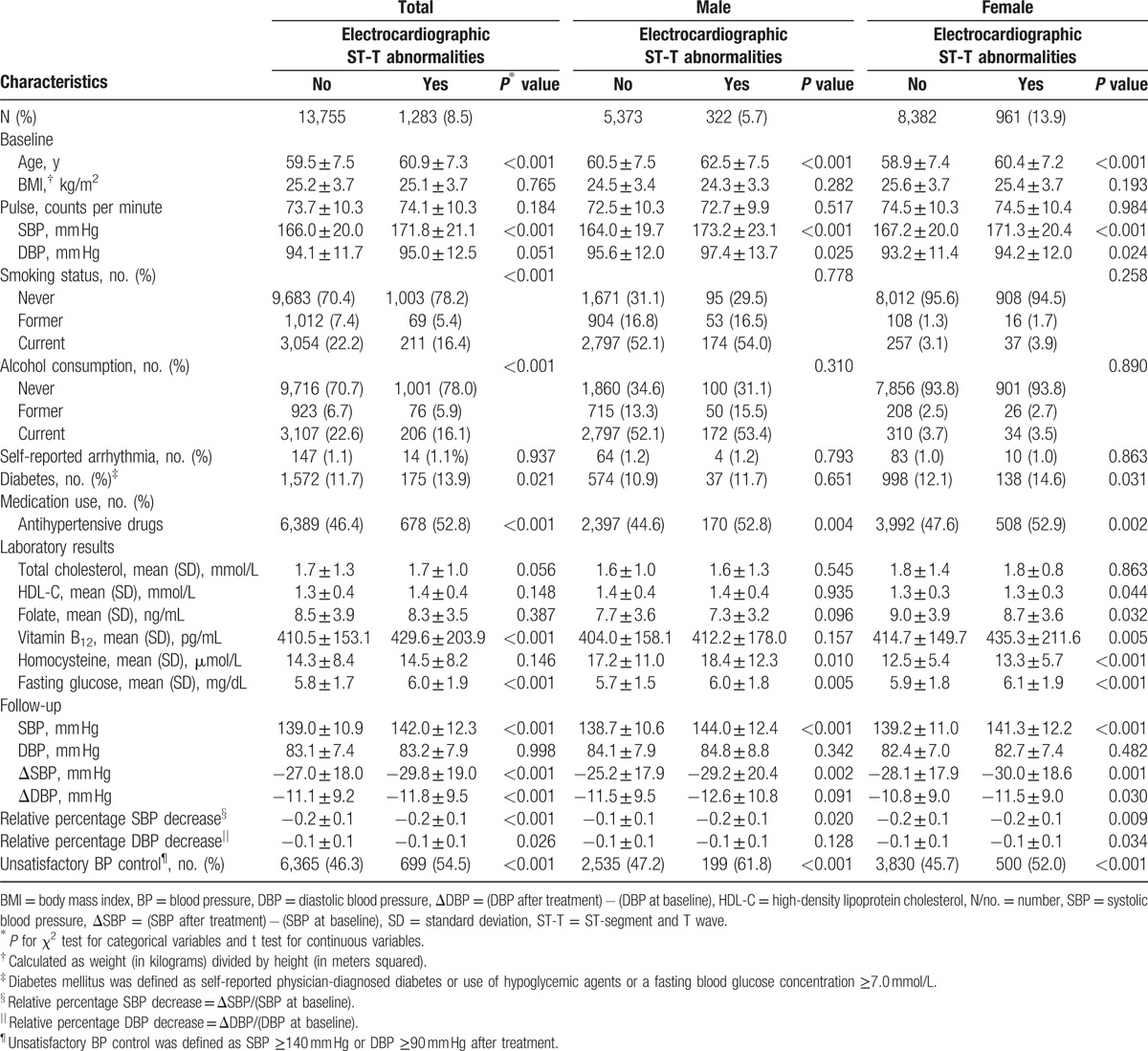
Baseline and follow-up characteristics of the study participants.

The presence and absence of nonspecific ST-T abnormalities significantly differed among related demographic and clinical variables (Table 1). This trend potentially affected the outcome (failure to achieve BP treatment goals) (Table 1). The independent relationship between the outcome and the presence and/or absence of nonspecific ST-T abnormalities was examined following adjustment for the possible effects of the following parameters: center, treatment group, self-reported arrhythmia, antihypertensive drug use, β-blocker use, smoking status and alcohol consumption, baseline SBP and DBP, gender, age, pulse rate, glucose, homocysteine, folate, vitamin B_12_, total cholesterol level, high-density lipoprotein cholesterol, and BMI (Table 2). The unsatisfactory BP control rate of the total population was significantly higher in the ST-T abnormal group (odds ratio [OR] 1.20, 95% confidence interval [CI] [1.06, 1.36], *P* = 0.005]), whereas with regard to the stratified categories significantly different results were obtained in male (OR 1.51, 95% CI [1.17, 1.94], *P* = 0.002) and not in female subjects (OR 1.11, 95% CI [0.96, 1.28], *P* = 0.160).

**Table 2:**
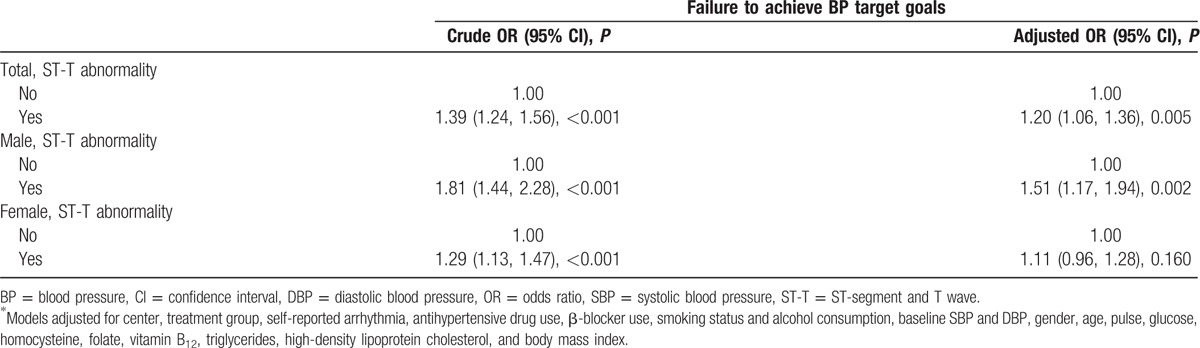
Association between baseline electrocardiographic ST-T abnormality and failure to achieve blood pressure treatment goals.

The associations between baseline electrocardiographic ST-T abnormalities and changes in BP following treatment are shown in Table 3. Following adjustment for the covariables described in Table 2, the patients with nonspecific ST-T abnormalities exhibited significantly lower changes in SBP and DBP (β 1.39, 95% CI [0.82, 1.96], *P* < 0.001, and β 0.50, 95% CI [0.19, 0.82], *P* = 0.002, respectively). In addition, these patients revealed lower values in the relative percentage decrease in SBP and DBP (β 0.80, 95% CI [0.45, 1.15], *P* < 0.001, and β 0.54, 95% CI [0.20, 0.88], *P* = 0.002, respectively). Importantly, the aforementioned differences were noted in male and not female subjects.

**Table 3:**
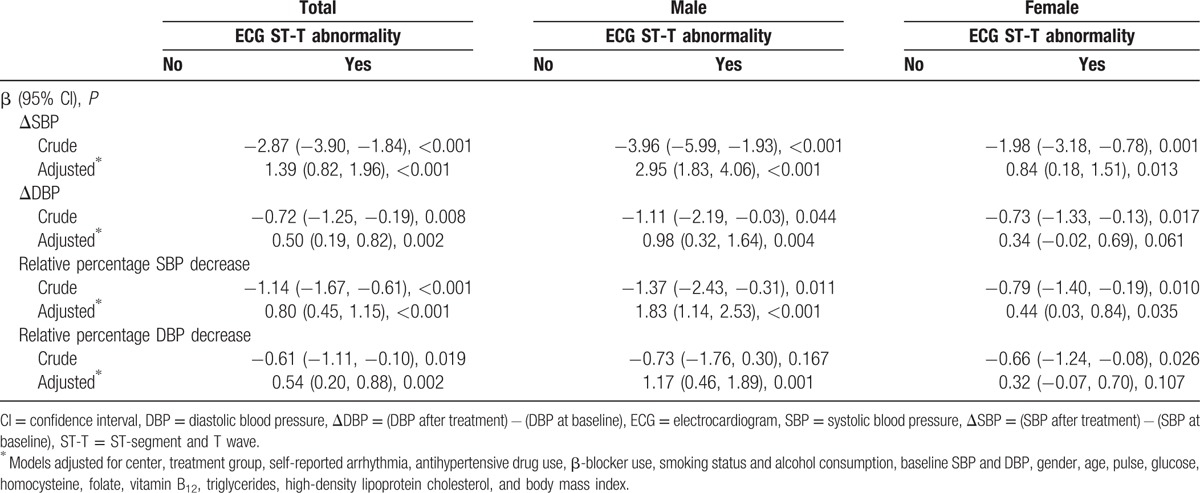
Association between baseline electrocardiographic ST-T abnormality and change of blood pressure under treatment.

Table 4 indicates the results of the multivariate logistic regression models regarding the assessment of the impact of electrocardiographic ST-T abnormalities on unsatisfactory BP control in the subgroup analyses. Following adjustment for the confounders described in Table 2, the differences were further observed between the following subgroups: patients with enalapril and folic acid treatment, patients from the Jiangsu center, male subjects, older patients at an age ≥60 years, middle and high baseline SBP tertiles, high baseline DBP tertile, low BMI, and comorbid diabetes. The unsatisfactory BP control rate for the aforementioned subjects was significantly greater in the presence of nonspecific ST-T abnormalities. Among these patients notable differences were noted in male subjects (OR 1.51, 95% CI [1.17, 1.94], *P* = 0.002) and subjects with comorbid diabetes (OR 1.47, 95% CI [1.04, 2.07], *P* = 0.029).

**Table 4:**
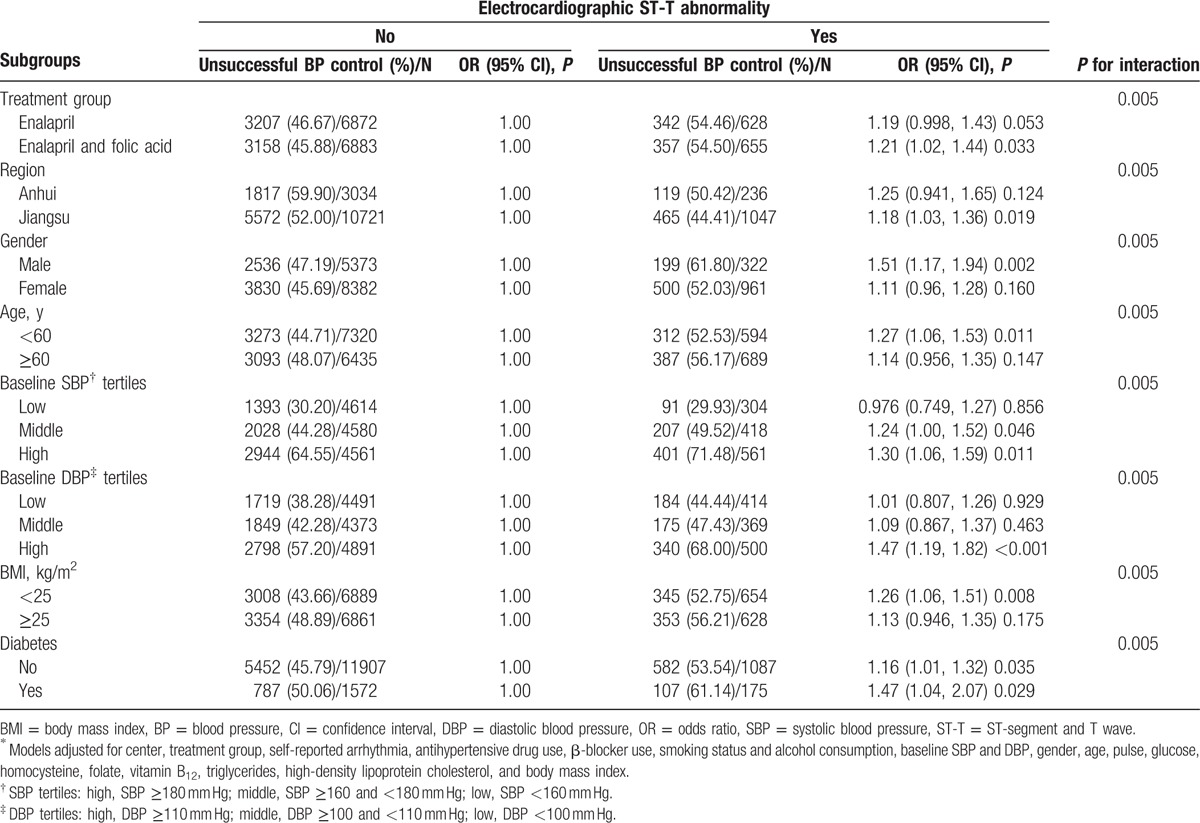
Multivariate logistic regression^∗^ evaluating the impact of electrocardiographic ST-T abnormality on unsatisfactory BP control in subgroup analyses.

## 4 Discussion

The major advantage of the present study was the large sample size derived from the CSPPT. The CSPPT was a large randomized trial conducted in adult subjects with hypertension in China without a history of stroke or myocardial infarction. The results of the trial demonstrated that enalapril–folic acid therapy significantly reduced the relative risk of an initial stroke incident by 21% compared with enalapril monotherapy. Given the well-characterized population, the standardized assessment of ECGs using the MC criteria, and the 4.5-year longitudinal follow-up period, the CSPPT study provided a unique opportunity to assess the prevalence of nonspecific ST-T changes in order to identify differences between genders and to explore whether nonspecific ST-T changes were associated with increased risk of unsatisfactory BP control. The study further provided insight regarding the potential predictive value of unsatisfactory BP control.

In the present study, the rate for the achievement of target BP levels of the total population was 53.0% and that was considerably greater compared with that previously reported in a national survey among adult participants in China (27.4% among treated hypertensive participants).^[5]^ The possible explanations of the aforementioned findings may be the rigorous design of the CSPPT and the strict follow-up schedule applied by trained research staff and physicians, which ensured high adherence of patients to the prescribed treatment regimen. The control rate of the present study conducted in China was similar to that noted in 2 studies conducted in America (55.8%, result from the HATT study)^[10]^ and Italy (55.6%–66.3%, analysis of a large database).^[11]^

The current study demonstrated that nonspecific ST-T changes in the ECG of hypertensive patients can be used for the identification of a greater risk of unsatisfactory BP control, notably in male participants and patients with diabetes mellitus. The prevalence of nonspecific ST-T abnormalities was 8.5%, and the prevalence was greater in women (10.3%) and in patients with diabetes (13.9%). This finding is consistent with previous studies.^[7,12–16]^ The greater prevalence of nonspecific ST-T changes in women has been attributed to the interplay of a variety of factors of anatomic, structural, hormonal, autonomic, and genetic origin. High blood glucose is associated with comorbidities such as hypertension, hyperlipidemia, and a prothrombotic state that interact synergistically to promote cardiac changes. The latter changes in turn result in ECG abnormalities, including nonspecific ST-T changes.

As depicted in Table 1, SBP was significantly greater in hypertensive patients with baseline nonspecific ST-T abnormalities, which was in agreement with a previously reported study by Vinyoles et al.^[17,18]^ As a result, nonspecific ST-T changes could be considered an early indicator of poor BP management. Following adjustment for the covariables, the current study indicated that hypertensive patients with baseline nonspecific ST-T abnormalities exhibited a higher rate of unsatisfactory BP control (OR 1.20, 95% CI [1.06, 1.36], *P* = 0.005) (Table 2), and significantly lower changes in SBP and DBP (Table 3). Lower degrees in relative percentage decreases of SBP and DBP following antihypertensive treatment were further noted (Table 3).

To date, there are no conclusive data regarding the association of nonspecific ST-T abnormalities with specific pathophysiologic mechanisms. It has been suggested that nonspecific ST-T changes might represent subclinical coronary artery disease, early left ventricular hypertrophy, increased left ventricular mass, or autonomic imbalance.^[7,19]^ The aforementioned parameters may increase the risk of unsatisfactory BP control, although further studies are required to clarify this hypothesis.

Recently, the majority of studies have demonstrated that nonspecific ST-T abnormalities are significantly associated with cardiovascular events and cerebrovascular accidents in hypertensive patients.^[20,21]^ In the present study, it was speculated that unsatisfactory BP control might play an intermediary role in this process. However, the exact mechanism of action remains unclear and further research work is required to address this issue.

In subgroup analyses, following adjustment for confounders, male subjects and the patients with comorbid diabetes with baseline nonspecific ST-T abnormalities exhibited notably greater rates of unsatisfactory BP control compared with the patients who exhibited no abnormalities. The greater risk of unsatisfactory BP control in male compared with female subjects may be related to the lack of the cardioprotective effect of estrogen, which has vasodilating and antioxidant properties, and is considered to influence cardiac natriuretic peptides via the renin–angiotensin system.^[22]^ In addition, high blood glucose increased the risk of unsatisfactory BP control, presumably via the association with cardiovascular target organ damage. Further studies are required to demonstrate these theories.

In conclusion, the identification of nonspecific ST-T abnormalities in the ECG of hypertensive patients is of considerable significance due to their potential application as markers for unsatisfactory BP control. Furthermore, the study is of particular importance as regards the majority of the Chinese population where BP is inadequately controlled. The findings may provide the basis for more intensive management of hypertensive patients who display electrocardiographic nonspecific ST-T abnormalities, notably for male subjects and/or patients with comorbid diabetes. In addition, it may be necessary for clinicians to prescribe more potent antihypertensive drugs during the course of hypertension treatment.

ECG machines are readily available in the majority of healthcare facilities and clinics in China. Consequently, ECG screening comprises a potentially accessible and affordable risk assessment tool in primary care settings in order to aid the management of BP control.

## 5 Limitations

One of the limitations encountered was the measurement of the cardiac activity by a single ECG during the baseline physical examination that prevented the exclusion of subsequent abnormalities during the follow-up period. Second, it is well established that nonspecific ST-T abnormalities have been associated with transient physiologic phenomena, namely ingestion of food, change in posture, and/or emotional distress. Additional postulated explanations regarding the nonspecific ST-T abnormalities include central nervous system lesions, abnormalities in the left ventricular wall motion in the absence of coronary artery disease, persistent juvenile pattern, electrolyte disturbances, use of drugs (i.e., digitalis, antiarrhythmic, and psychotropic drugs), and/or athletic ability.^[7]^ These processes limit the reproducibility of ST-T segment changes in successive ECG measurements. Third, BP measurements were not carried out at trough for the patients who received antihypertensive drugs and consequently the assessment of BP control could have been influenced to a certain extent. In the future, ambulatory BP monitoring may be a favorable selection for BP management and evaluation.
