# Nonspecific ST-T changes associated with unsatisfactory blood pressure control among adults with hypertension in China: Evidence from the CSPTT study: Erratum

**DOI:** 10.1097/MD.0000000000006811

**Published:** 2017-04-28

In the article, “Nonspecific ST-T changes associated with unsatisfactory blood pressure control among adults with hypertension in China: Evidence from the CSPTT study”,^[1]^ which appeared in Volume 96, Issue 13 of *Medicine*, the title appeared incorrectly. The title should have appeared as “Nonspecific ST-T changes associated with unsatisfactory blood pressure control among adults with hypertension in China: Evidence from the CSPPT study.”
