# Nonspecific ST-T changes associated with unsatisfactory blood pressure control among adults with hypertension in China: Evidence from the CSPPT study: Erratum

**DOI:** 10.1097/MD.0000000000008472

**Published:** 2017-10-27

In the article, “Nonspecific ST-T changes associated with unsatisfactory blood pressure control among adults with hypertension in China: Evidence from the CSPPT study”^1^, which appeared in Volume 96, Issue 17 of *Medicine*, the author requested changes to tables 2–4.

**Table 2:**
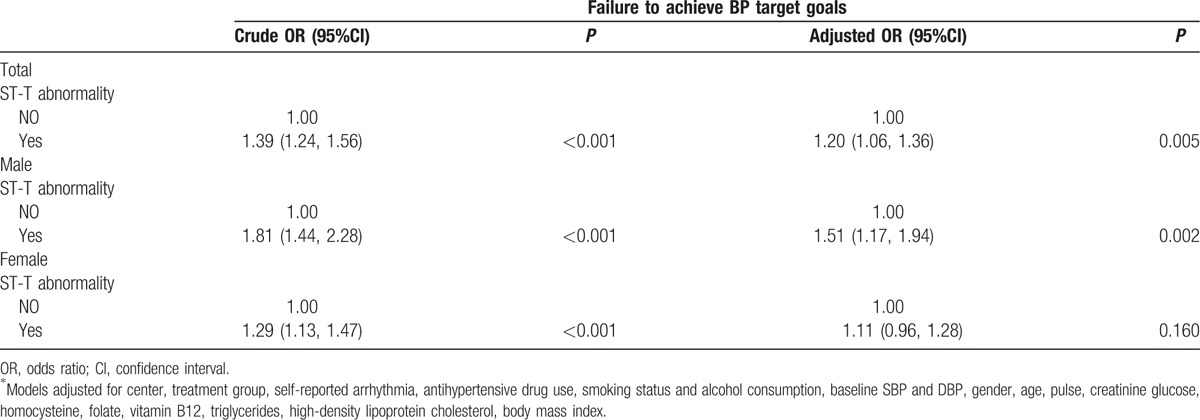
Association between baseline electrocardiographic ST-T abnormality and failure to achieve blood pressure treatment goals.

**Table 3:**
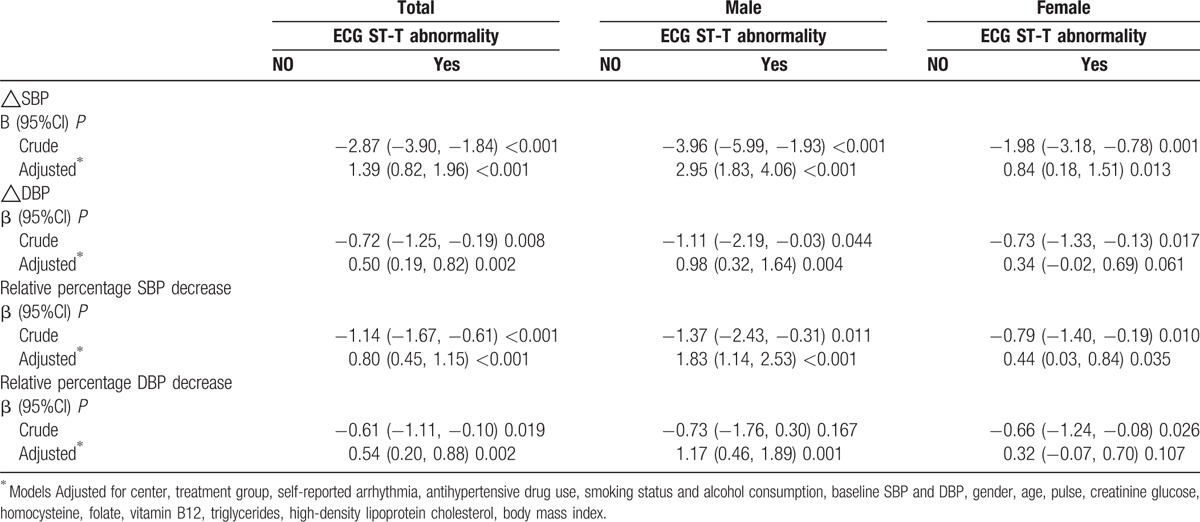
Association between baseline electrocardiographic ST-T abnormality and change of blood pressure under treatment.

**Table 4:**
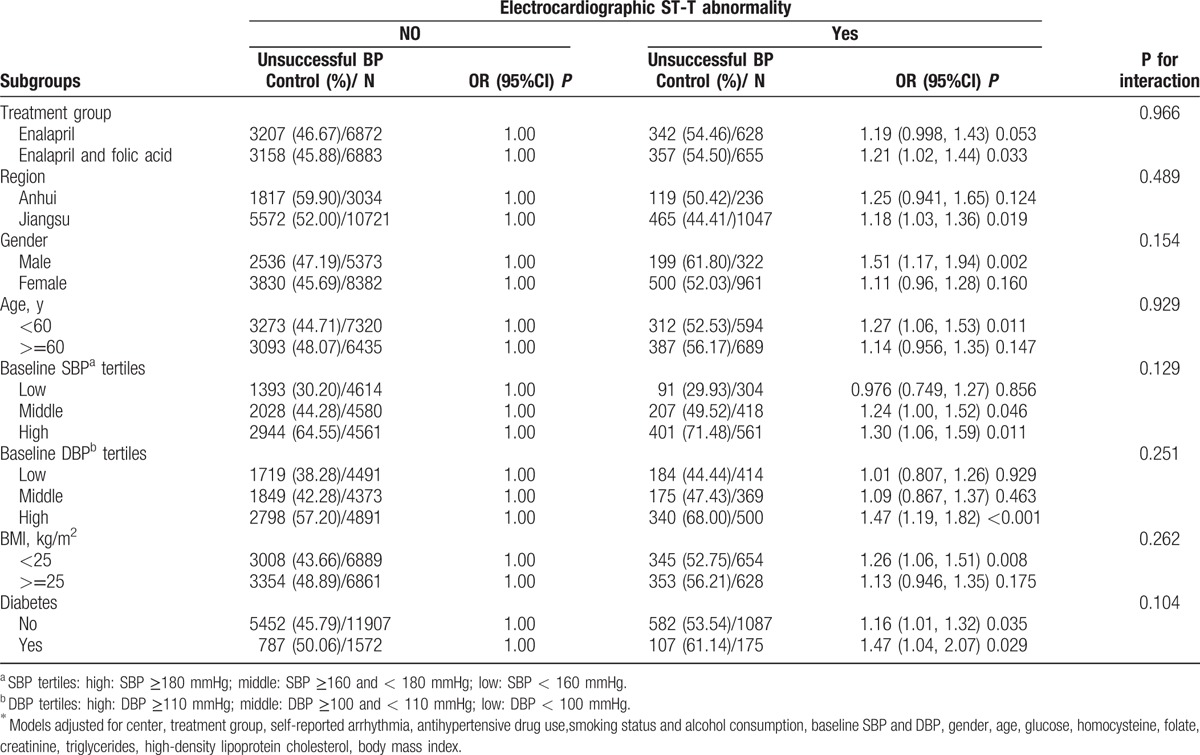
Multivariate logistic regression^∗^ evaluating the impact of electrocardiographic ST-T abnormality on unsatisfactory BP control in subgroup analyses.

In tables 2–4, the authors have added the adjustment variable “creatinine” and removed “β-Blockers use” and the text should read “Models adjusted for center, treatment group, self-reported arrhythmia, antihypertensive drug use, smoking status and alcohol consumption, baseline SBP and DBP, gender, age, glucose, homocysteine, folate, creatinine, triglycerides, high-density lipoprotein cholesterol, body mass index.”

In table 4, the ‘P for interaction’ of each variable has been adjusted. The original figure ‘P for interaction’ incorrectly listed 0.005 for each variable.
